# A novel model to predict mental distress among medical graduate students in China

**DOI:** 10.1186/s12888-021-03573-9

**Published:** 2021-11-15

**Authors:** Fei Guo, Min Yi, Li Sun, Ting Luo, Ruili Han, Lanlan Zheng, Shengyang Jin, Jun Wang, Mingxing Lei, Changjun Gao

**Affiliations:** 1 Department of Anesthesiology, The Second Affiliated Hospital of Air Force Medical University, Xi’an, 710038 People’s Republic of China; 2 Institute of Medical Information, Chinese Academy of Medical Sciences/Peking Union Medical College, Beijing, People’s Republic of China; 3 Department of Obstetrics and Gynecology, the Second Xiangya Hospital of Central South University, Changsha, People’s Republic of China; 4 Plastic Surgery Hospital, Chinese Academy of Medical Sciences and Peking Union Medical College, Beijing, People’s Republic of China; 5 Department of Anesthesiology, Shaanxi Provincial Armed Police Hospital, Xi’an, People’s Republic of China; 6 Chinese PLA Medical School, Beijing, 100853 People’s Republic of China; 7 Department of Orthopedic Surgery, National Clinical Research Center for Orthopedics, Sports Medicine & Rehabilitation, Chinese PLA General Hospital, Beijing, People’s Republic of China

**Keywords:** Mental distress, Prediction model, Medical graduate student

## Abstract

**Background:**

Poor mental health was reported among medical graduate students in some studies. Identification of risk factors for predicting the mental health is capable of reducing psychological distress among medical graduate students. Therefore, the aim of the study was to identify potential risk factors relating to mental health and further create a novel prediction model to calculate the risk of mental distress among medical graduate students.

**Methods:**

This study collected and analyzed 1079 medical graduate students via an online questionnaire. Included participants were randomly classified into a training group and a validation group. A model was developed in the training group and validation of the model was performed in the validation group. The predictive performance of the model was assessed using the discrimination and calibration.

**Results:**

One thousand and fifteen participants were enrolled and then randomly divided into the training group (*n* = 508) and the validation group (*n* = 507). The prevalence of severe mental distress was 14.96% in the training group, and 16.77% in the validation group. The model was developed using the six variables, including the year of study, type of student, daily research time, monthly income, scientific learning style, and feeling of time stress. The area under the receiver operating characteristic curve (AUROC) and calibration slope for the model were 0.70 and 0.90 (95% CI: 0.65 ~ 1.15) in the training group, respectively, and 0.66 and 0.80 (95% CI, 0.51 ~ 1.09) in the validation group, respectively.

**Conclusions:**

The study identified six risk factors for predicting anxiety and depression and successfully created a prediction model. The model may be a useful tool that can identify the mental status among medical graduate students.

**Trial registration:**

No.ChiCTR2000039574, prospectively registered on 1 November 2020.

**Supplementary Information:**

The online version contains supplementary material available at 10.1186/s12888-021-03573-9.

## Background

Mental distress, characterized by a broad range of behavioral and psycho-physiological symptoms, is a mental health problem often relating to mental disorders, such as depression and anxiety [1]. A global prevalence of depression among medical students was up to 28.0% [2] and the prevalence of anxiety was 33.8% among medical students, which is significantly higher than the general population [3]. Moreover, a recent study reported a high prevalence of depression (29%) and anxiety (21%) among Chinese medical students [4]. All these data alarmed that a large number of medical students around the world were experiencing severe mental distress which could impair their psychosocial functioning, physical health, professional and academic performance, and ultimately cause serious consequences including divorce, crime, self-harm, and suicide tendency [5–9]. Meanwhile, a large body of literature revealed that mental distress was the largest cost driver of the global economic burden of non-communicable diseases [10]. Therefore, it is an urgent issue to find causes, preventions, and solutions to mental distress.

Identification of risk factors for anxiety and depression is capable of helping early detection and intervention and preventing more serious consequences. As it indicated in recent studies, medical students were at high risks of mental distress, which were contributed to severe academic, psychological, and emotional stress, including academic demands, workload, pressure from teachers and parents, financial burden and worry about the future [11–13]. Mental distress, including depression and anxiety, can be evaluated by the Symptom Check List-90 (SCL-90), Beck Depression Inventory (BDI), to name just a few. However, the above scales were primarily used to evaluate the mental status of the general population by professional medical workers. In addition, although these scales were beneficial in measuring and diagnosing mental distress, they cannot predict the occurrence of unhealthy mental status in advance. Notably, a previous study indicated that the role of inadequate self-awareness about one’s mental health concerns was a barrier to reaching out for professional help [14]. It highlighted the importance of expanding the range of factors beyond commonly studied concepts like the demand-control model and the effort-reward imbalance model [15].

Therefore, this study aimed to identify potential factors associating with mental health and further develop a novel model to predict the probability of mental distress, especially among medical graduate students. We speculated that the formula could present the relationships between mental distress and the potential risk factors, and the contributions of these factors could be quantified by assigning scores and correlation coefficients in the formula.

## Methods

### Study design and sample size estimation

A cross-sectional survey was conducted from November 2020 to December 2020. We designed a questionnaire (Additional file 1) after thoroughly reviewing available literature and discussing it with some medical graduate students, investigators, and senior professors. After discussion, we revised and further improved the questionnaire based on valuable suggestions. The online questionnaire was distributed through the instant communication tools, including telephone messages, emails, Opening I CQ [Seek You] (OICQ) software, and WeChat software, via a nonprobability snowball sampling strategy [16] focusing on recruiting medical graduate students all around China. The initial participants (seeds) in this snowball sampling were medical graduate and postgraduate students with good academic performance. In detail, the majority of them were working and studying in the Peking Union Medical College, Xiangya School of Medicine, and West China Medical Center, all of which had a great reputation in the medical field of China. Besides, they had long-term scientific contacts with the researchers. We stopped the survey a month later after collecting enough participants since the sample was not increasing at that time and enough participants were collected. Moreover, during the investigation, notably, we did not take financial compensation strategies to attract participants to take part in the survey because this might contribute to selection bias. Participants were totally voluntarily to take part in the survey. Informed consents were obtained from all participants before completing the questionnaire.

The questionnaire contains about 20 questions and it takes 3 to 5 min to complete. This study was conducted online anonymously without obtaining participant’s any personally identifiable information. All valid information including associated device, IP address, and answers for each question was collected anonymously, and then we constructed a basic database about the mental distress among medical graduate students by automatic collation and graphical representation for each question. We excluded those who were post-doctors and reported a previous diagnosis with depression or anxiety in the hospital. The participants were then randomly divided into the training group and the validation group. The training group was used to develop a formula to calculate the prevalence of mental distress among medical graduate students in China. Meanwhile, internal validation of the formula was performed in the validation group.

For the estimation of sample size, we took the prevalence of 28% [17] for mental distress from a study performed among Chinese graduate students and 95% certainty and ± 5% margin of error. Considering 10% of non-response rate, the sample size was estimated to be about 344.

### Ethics approval and study registration

The aims and procedures of the study were reviewed and approved by the Research Ethics Committee of Plastic Surgery Hospital of Chinese Academy of Medical Science (No: 2020157). The study was also registered at the Chinese Clinical Trial Registry (Registration number: ChiCTR2000039574). All procedures used complied with the ethical principles on human experimentation and with the Helsinki Declaration of 1975 as revised in 2008.

### Instruments

Potential risk factors and the main observation parameter (severe mental distress) in this study were collected by the questionnaire consisting of sociodemographic characteristics, academic performance, the incumbency of a tutor, and psychological evaluation. Sociodemographic characteristics included age, year of study, major and university location (provincial capital or other cities), marital status, and monthly income. The academic performance included pursued degree, double first-rate university, type of student, types of research, daily research time, scientific learning style, number of research projects and published papers, feeling of time stress (ranging from 1 to 7, 1 means none, 2–3 were mild, 4–5 were moderate, and 6–7 were severe). Incumbencies of a tutor mean that the tutor has administration position in corresponding departments, Chinese Academy of Sciences (CAS), Chinese Academy of Engineering (CAE), or national academic organizations. Whether the tutor won a bid of National Natural Science Foundation of China (NSFC) or not within the past 5 years was also collected.

Psychological evaluation was based on the Generalized Anxiety Disorder Scale-7 (GAD-7) and Patient Health Questionnaire-9 (PHQ-9). GAD-7 is a 7-item self-report scale to measure anxiety symptoms [18]. In this scale, each question was designed to assess the frequency of anxiety, with scores ranging from 0 (never) to 3 (daily). The total score is 0 to 21, coming from the sum of the values for each item. The reported Cronbach’s α coefficient of the GAD-7 among Chinese participants is 0.92 [19]. The Cronbach’s α of the GAD-7 was 0.94 in the present study. PHQ-9 is a 9-item scale based on criteria for depressive disorders in the Diagnostic and Statistical Manual of Mental Disorders (DSM-IV) [20, 21] to measure depression symptoms. Each item scores from 0 to 3 according to the increasing intensity of symptoms. The PHQ-9 had a Cronbach’s α of 0.86 [22]. The Cronbach’s α of the PHQ-9 was 0.92 in the present study. The Cronbach’s α of the whole questionnaire was 0.83. When the items of GAD-7 and PHQ-9 were excluded in analysis, the Cronbach’s α of the questionnaire was 0.52. The severe mental distress in this study was defined as the sum of GAD-7 and PHQ-9 scores ≥30.

### Formula development

The Least Absolute Shrinkage and Selection Operator (LASSO) logistic model was used to investigate potential predictors according to computing efficient model descriptions of nonlinear systems. Variables with a coefficient value of more than 0.01 were included in the formula. The estimates used to develop the formula were obtained after the included variables re-entered the multiple logistic regression analysis. Finally, a formula was developed: P(Y = 1) = e^intercept + ax1 + bx2 + … + ixn^ / (1+ e^intercept + ax1 + bx2 + … + ixn^). In the formula, a, b, …, and i were the estimates, *x*1 to *xn* were the included variables, and P(Y = 1) indicated the predicted probability of severe mental distress among medical graduate students. The predicted probability indicated that the risk probability of severe mental distress was calculated from the developed formula based on the included factors.

### Validation of the formula

Internal validation of the formula was performed with the discrimination and calibration ability in the training and validation group. The discrimination ability of the formula was to separate students who developed mental distress from those who did not. The calibration ability of the formula was the consistency to observe and predict the prevalence of severe mental distress. The AUROC, which is the probability of concordance between the predicted and observed prevalence of mental distress among medical graduate students, was also calculated to measure the predictive effects of the formula’s discrimination ability. An AUROC of more than 0.7 indicates good predictive performance and 0.8 or above indicates excellent predictive performance. Furthermore, the discrimination ability of the formula was evaluated by the discrimination slope that was defined as the difference between the mean predicted risk probability with and without mental distress among medical students. We plotted deciles of the predicted probability of severe mental distress against the observed risk of severe mental distress in each decile and fitted a smooth line. Ideally, the slope of the fitted smooth line would be close to 1 and intercepts close to 0. Besides, the Hosmer-Lemeshow goodness-of-fit test was used to evaluate the formula’s calibration ability. A *P*-value of more than 0.05 from this test indicates good agreement between the predicted matrix and the observed matrix.

### Statistical analysis

Descriptive statistics were tabulated for the overall sample and stratified by the type of answers received. Continuous variables were presented as mean ± standard deviation (SD), while frequency and percentage were calculated for categorical variables. The potential risk factors were screened by the Least Absolute Shrinkage and Selection Operator (LASSO) method. Then, variables with a coefficient value > 0.01 were included in a multinomial logistic regression model to explore the estimates of the included variables in the formula. Statistical significance was set at *P* < 0.05 level with two-sided tests. Statistical analyses were performed using SAS 9.2 (SAS Institute Inc., Cary, NC) for Windows XP.

## Results

### Basic characteristics

**Fig. 1:**
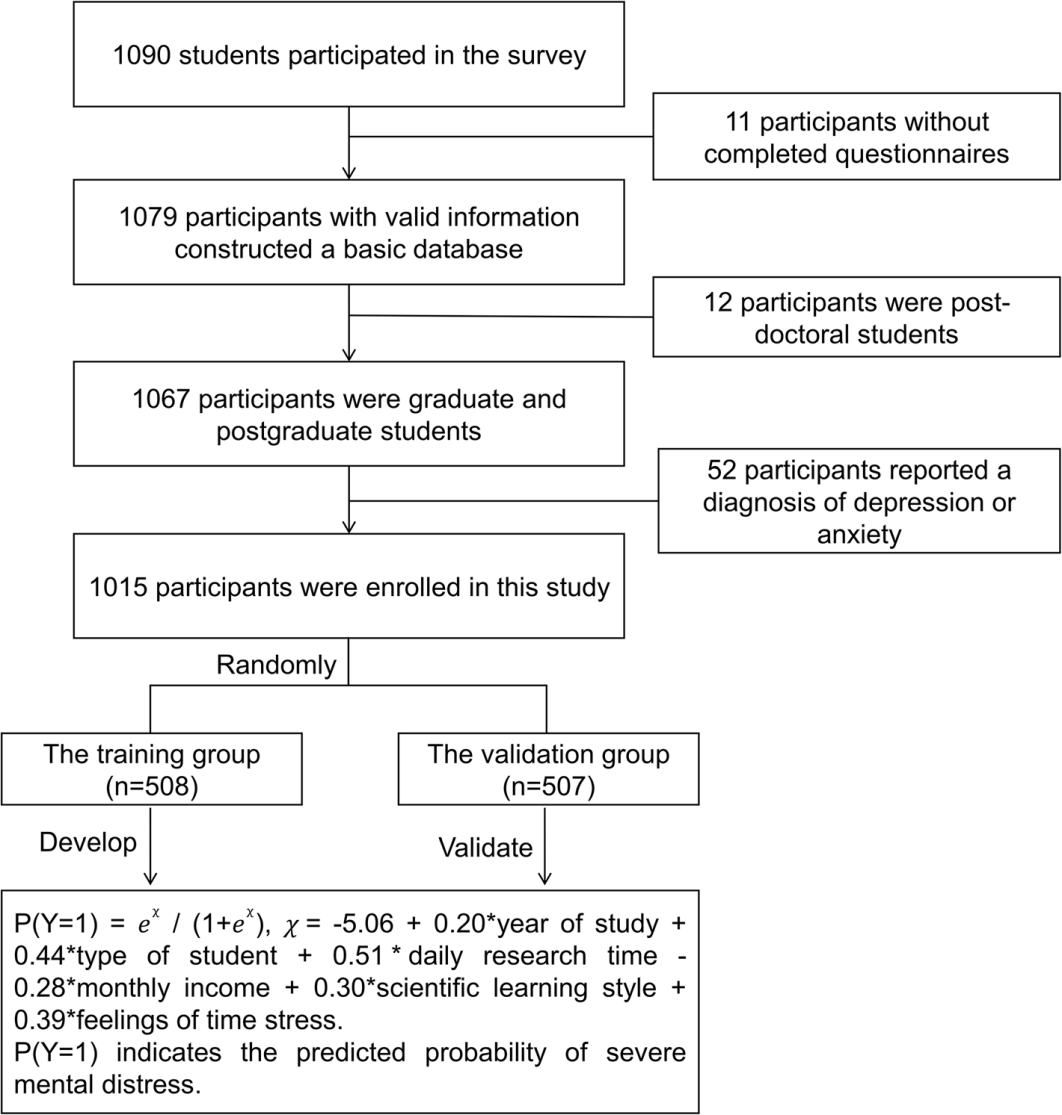
Study profile

One thousand and ninety students participated in this study, and 11 of them did not complete the questionnaire. Thus, a basic database was constructed by 1079 students with valid information. After excluding 12 post-doctors and 52 students who have reported diagnoses of depression or anxiety, 1015 participants were finally enrolled. Figure 1 shows the study profile.

Basic characteristics of participants in the training and validation groups are presented in Table 1. The mean age of the enrolled students was 25 years and more than half of all participants were single. The majority of the students were pursuing a master’s degree in clinical fields with a lower monthly income. A large proportion of the students were from the ‘Double First-rate’ university (74.41% in the training group and 76.73% in the validation group). About 70% of the students had participated in 1 to 3 research projects and had a longer daily working time. However, most of them (67.68%) have never published an academic paper in English or Chinese.

**Table 1:** Comparisons of basic characteristics between the training group and validation group

Characteristics	Training group (508)	Validation group (507)	*P*
Age (mean, years)	25.56 ± 3.09	25.32 ± 3.26	0.22
Year of study			0.04
First-year	196	215	
Second-year	178	183	
Third-year	109	100	
Fourth-year	11	6	
In deferment period	14	3	
Major			0.28
Obstetrics and gynecology	29	20	
Surgery	96	118	
Internal Medicine	138	122	
Basic Medicine	35	32	
Others	210	215	
University located at Beijing			0.68
Yes	51	47	
No	457	460	
Marital status			0.09
Single	268	245	
In love	187	184	
Married without childbearing	20	35	
Married and completed childbearing	33	43	
Monthly income (CNY)			1.00
<1000	113	115	
1000 ~ 3000	305	303	
3000 ~ 5000	41	40	
>5000	49	49	
Pursued degree			0.73
Graduate	385	389	
Postgraduate	123	118	
Double first-rate university			0.39
Yes	378	389	
No	130	118	
Type of student			0.40
Research-oriented	280	266	
Professional oriented	228	241	
Types of research			0.28
Basic scientific research	194	169	
Clinical research	164	171	
Both	117	138	
Uncertain	33	29	
Daily research time (Hours)			0.64
1–5	215	233	
6–10	153	142	
11–15	126	116	
≥ 16	14	16	
Number of published academic papers			0.69
0	338	349	
1–2	119	111	
3–4	29	31	
≥ 5	22	16	
First author paper, SCI index			0.27
Yes	122	107	
No	386	400	
Number of participating projects			0.07
0	130	143	
1–3	357	355	
≥ 4	21	9	
Scientific learning style			0.34
Guiding be others	313	327	
Self-study	195	180	
Feelings of time stress (Range from 1 to 7, 1 is none, 2–3 mild, 4–5 moderate, 6–7 severe)			0.31
1 (None)	16	18	
2–3 (Mild)	45	52	
4–5 (Moderate)	159	132	
6–7 (Severe)	288	305	
The tutor is director of the department			0.59
Yes	289	280	
No	219	227	
Tutor is academician of the CAS and CAE			1.00
Yes	3	3	
No	505	504	
Tutor hold position in the national academic organizations			0.03
Yes	61	40	
No	447	467	
Tutor won a bid of NSFC within 5 years			0.44
Yes	356	344	
No	152	163	
GAD-7 (mean)	8.75 ± 5.41	8.85 ± 5.39	0.77
PHQ-9 (mean)	8.65 ± 6.17	8.90 ± 5.94	0.29
Severe mental distress			0.43
Yes	76	85	
No	432	422	

*Abbreviations*: *SCI* Science Citation Index, *CNY* Chinese Yuan, *CAS* Chinese Academy of Sciences, *CAE* Chinese Academy of Engineering, *NSFC* National Natural Science Foundation of China, *GAD-7* Generalized Anxiety Disorder Scale-7, *PHQ-9* Patient Health Questionnaire-9

Notes: Severe mental distress was defined as the sum of GAD-7 and PHQ-9 scores ≥30

**Fig. 2:**
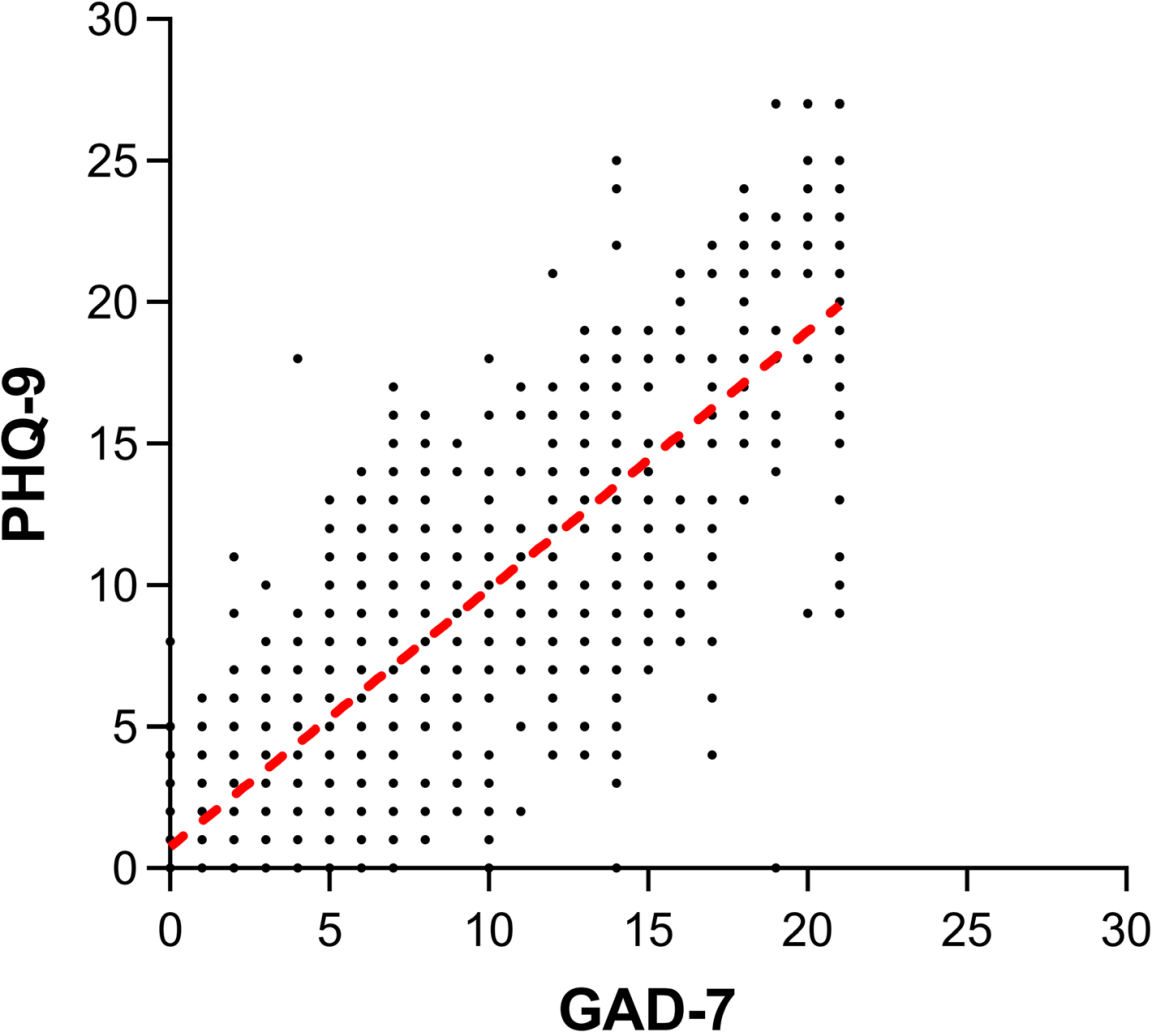
Correlations between GAD-7 and PHQ-9. The curve can be fitted with a line: y = 0.91x + 0.75 (x indicates the GAD-7, y indicates the PHQ-9)

More than 55% of participant’s tutors were leaders of the department among the two groups and most of them had won the bid of the National Natural Science Foundation of China in past 5 years. According to the defined cut-offs of GAD-7 and PHQ-9, the prevalence of severe mental distress was 14.96% among the enrolled students. The relationship between the GAD-7 and PHQ-9 shows a good association in Fig. 2.

### The formula development

After data extraction, the 1015 participants were randomly divided into the training group (*n* = 508) and the validation group (*n* = 507). In the training group, we found that seven predictors, including the year of study, type of student, types of research, daily research time, monthly income, scientific learning style, and feeling of time stress, were significantly associated with severe mental distress. The types of research were not included in the formula due to the low coefficient value (< 0.01). Finally, the left six variables were included in the formula and the corresponding estimates were obtained from the multiple logistic regression analysis (Table 2). A formula was successfully developed as follows: P(Y = 1) = e^x^ / (1+ e^x^), x = − 5.06 + 0.20*Year of study + 0.44*Type of student + 0.51*Daily research time - 0.28*Monthly income + 0.30*Scientific learning style + 0.39*Feelings of time stress. P(Y = 1) indicates the predicted probability of severe mental distress. The score in each variable was assigned according to the original dataset. An example of how to use the formula was given in the discussion section.

**Table 2:** The significant characteristics are included in the model and corresponding estimates

Characteristics	Estimates^a^	Assigned score
Intercept	−5.06	
Year of study		
First-year	0.20	1
Second-year		2
Third-year		3
Fourth-year		4
In deferment period		5
Type of student		
Research-oriented	0.44	1
Professional oriented		2
Daily research time (Hours)		
1–5	0.51	1
6–10		2
11–15		3
≥ 16		4
Monthly income (CNY)		
<1000	−0.28	1
1000 ~ 3000		2
3000 ~ 5000		3
>5000		4
Scientific learning style		
Guiding by others	0.30	1
Self-study		2
Feelings of time stress		
1 (None)	0.39	1
2–3 (Mild)		2
4–5 (Moderate)		3
6–7 (Severe)		4

*Abbreviation*: *CNY* Chinese Yuan

Notes: Seven predictors were identified by the LASSO method to be associated with the prevalence of severe mental distress significantly. Estimates were calculated using the logistic regression model. Severe mental distress was taken as Y in the model. Six variables were included in the model according to the coefficient values (value>0.01)

The formula: P(Y = 1) = *e*^*x*^ / (1+ *e*^*x*^). *x* = − 5.06 + 0.20* Year of study + 0.44*Type of student + 0.51*Daily research time - 0.28*Monthly income + 0.30*Scientific learning style + 0.39*Feelings of time stress. P(Y = 1) indicates the predicted probability of severe mental distress

### Internal validation of the formula

**Fig. 3:**
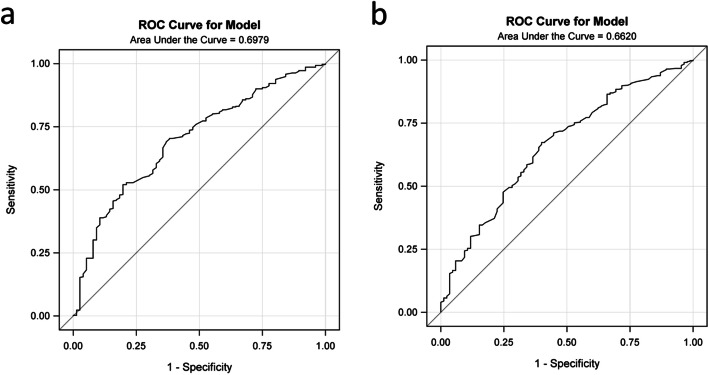
**a** ROC curve for the developed model in the training group. **b** ROC curve for the developed model in the validation group

**Fig. 4:**
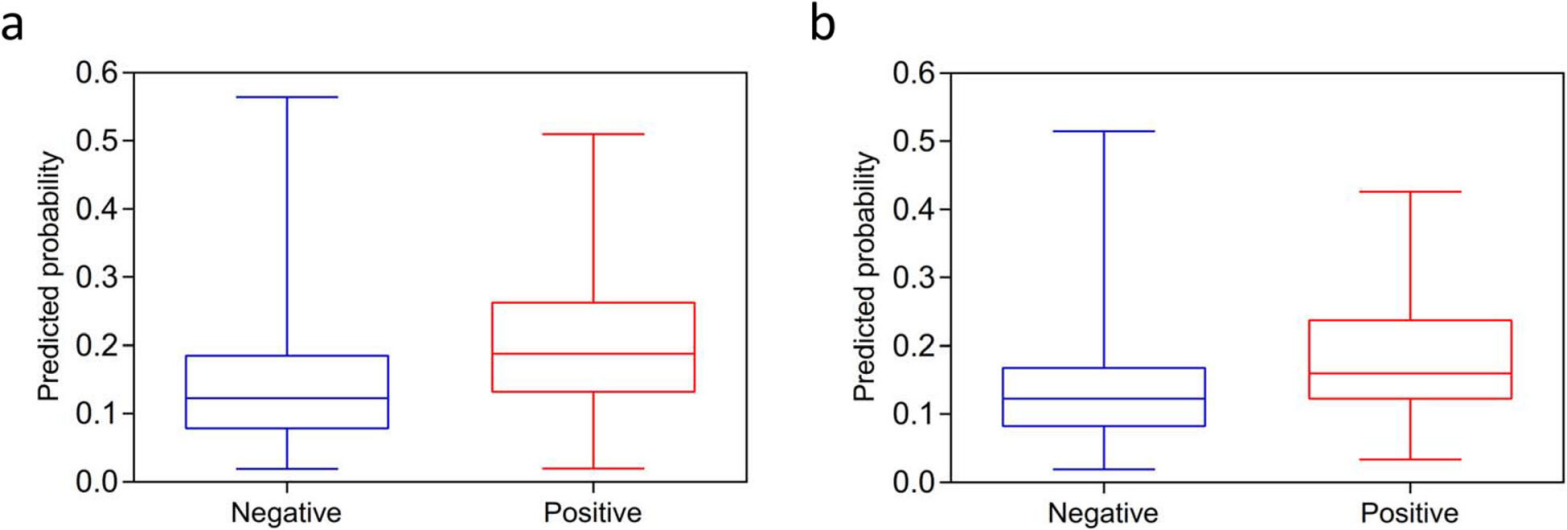
**a** Discrimination slope of the developed model in the training group (Slope = 0.06, Negative:0.14 vs Positive:0.20, *P*<0.001). **b** Discrimination slope of the developed model in the validation group (Slope = 0.04, Negative:0.13 vs Positive:0.17, *P*<0.001)

The formula presented relative good discrimination ability exactly as the AUROC was 0.70 in the training group and 0.66 in the validation group (Table 3 and Fig. 3a and b). The corresponding discrimination slopes were 0.06 (95% CI: 0.04 ~ 0.08, *P* < 0.001) and 0.04 (95% CI: 0.02 ~ 0.06, P < 0.001) (Fig. 4a and b), respectively. The correct classification rates were 82.30% in the training group, and 81.30% in the validation group. Comparing sensitivity and specificity between the training group and validation group, they were 11.80% vs. 4.70 and 94.70% vs. 96.70%, respectively. The false-positive was more than 70% and false-negative rates were both below 20% in the two groups, which indicated high sensitivity of the formula.

**Table 3:** The effective performances of the model in the validation group

Discrimination ability	AUROC	Slope^a^	95% CI	CCR	Sensitivity	Specificity	False POS	False NEG
Training group	0.70	0.06	0.04 ~ 0.08	82.30%	11.80%	94.70%	71.90%	14.10%
Validation group	0.66	0.04	0.02 ~ 0.06	81.30%	4.70%	96.70%	77.80%	16.60%
Calibration ability	Slope^b^	95% CI	X-intercept	95% CI	Y-intercept	95% CI	P^c^	
Training group	0.90	0.65 ~ 1.15	−0.02	−0.09 ~ 0.03	0.02	−0.03 ~ 0.06	0.82	
Validation group	0.80	0.51 ~ 1.09	−0.01	− 0.11 ~ 0.04	0.01	− 0.05 ~ 0.06	0.97	

*Abbreviations*: *AUROC* the area under the receiver operating characteristic curve, *CI* Confident interval, *CCR* Correct classification rate, *POS* Indicates positive, *NEG* indicates negative

^a^ indicates discrimination slope;

^b^ indicates calibration slope;

^c^ indicates Hosmer and Lemeshow Goodness-of-Fit Test

**Fig. 5:**
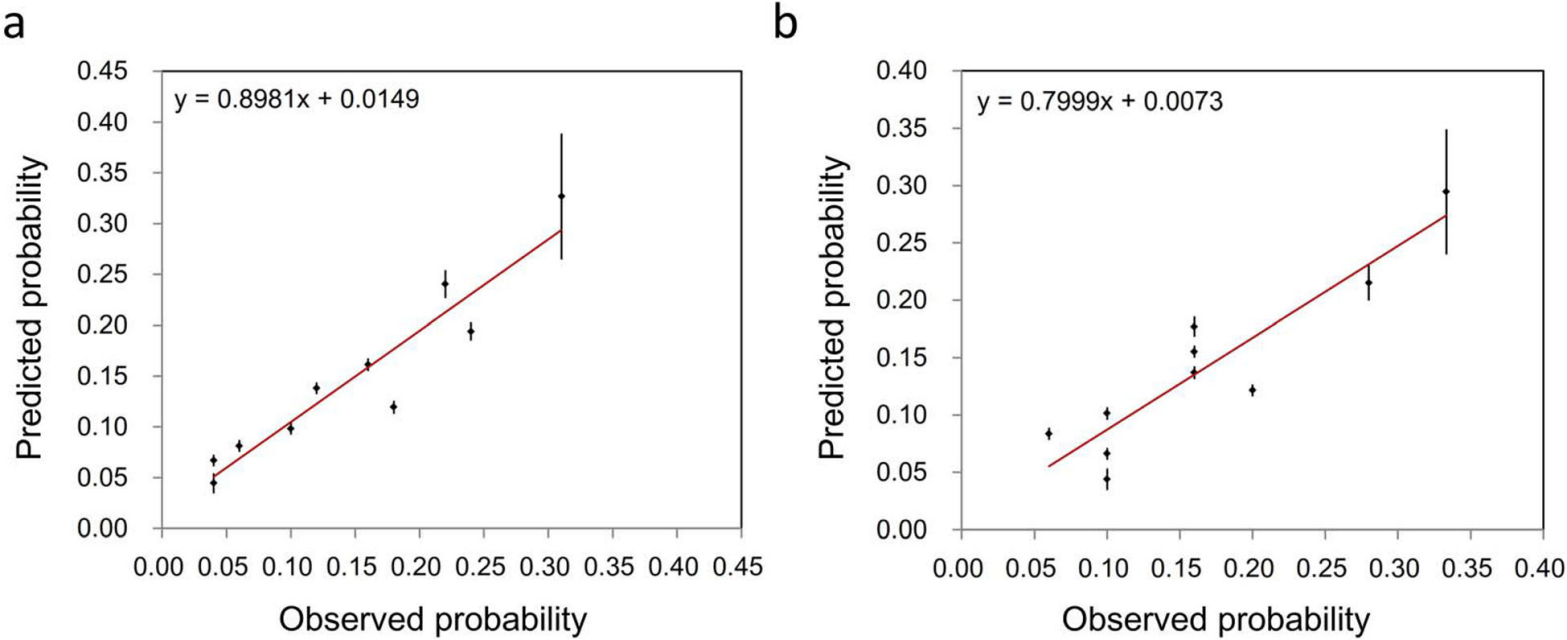
**a** Calibration slope of the developed model in the training group (Slope = 0.90, *P*<0.001). **b** Calibration slope of the developed model in the validation group (Slope = 0.80, *P*<0.001)

The calibration slope of the formula was 0.90 (95% CI: 0.65 ~ 1.15) in the training group and 0.80 (95% CI: 0.51 ~ 1.09) in the validation group (Table 3 and Fig. 5a and b). Because the X- and Y- intercepts were almost close to zero, the formula had good calibration ability.

### Classification of risk groups based on the developed model

According to the developed model, we divided participants into three risk groups (Table 4), including the low-risk group with a predicted probability of severe mental distress from 0 to 9.99%, the moderate risk group ranging from 10.00 to 19.99%, and the high-risk group with a predicted probability of severe mental distress of 20.00% or above. The actual probabilities of severe mental distress were similar to the predicted probabilities of severe mental distress between the three groups, which indicated that the classification was reproducible.

**Table 4:** Risk group classifications of the developed model

Groups	Number of students	Predicted value P(Y = 1)	Actual value P(Y = 1)	P^a^
Low risk (0.00% ~ 9.99%)	345	6.81%	6.96%	<0.001
Moderate risk (10.00% ~ 19.99%)	461	14.54%	16.49%	
High risk (20.00%~)	209	27.45%	29.19%	

Notes: P(Y = 1) indicates the rate of severe mental distress

^a^ indicates an actual rate of severe mental distress among the three risk groups

## Discussion

A formula was successfully developed to accurately assess the prevalence of severe mental distress after analyzing 1015 included participants. To better understand the sample characteristics, we compared the age and other basic characteristics of the sample with the general populations of medical students in China [23, 24] and there were no significant difference. The 1015 participants were used to develop and internally validate the formula which was simple and convenient since it consisted of six variables, including the year of study, type of student, daily research time, monthly income, scientific learning style, and feeling of time stress. All these included variables were readily available and accessible. After internal validation, the formula’s good discrimination and calibration ability was confirmed according to the results. The *P*-value of the Hosmer and Lemeshow Goodness-of-Fit Test was 0.97, which showed that the formula could be a relatively reliable prediction model among medical graduate students. We further divided participants into the low risk (0.00% ~ 9.99%), the moderate risk (10.00% ~ 19.99%), and the high risk (20.00%~) groups based on the predicted probability of the developed model. Participants in the high-risk group had an actual probability of severe mental distress of 29.19%, thus psychological counseling was recommended in those participants. The LASSO method was used to select potential risk factors in this study. Compared with other logistic regression models, it is a popular model-building procedure that shrinks a subset of coefficients to zero and could perform variable selection and estimation simultaneously [25].

Over the past decades, it was not uncommon to see that medical graduate students committed suicide due to mental distress, causing much grief and loss to society and their families. Studies have attempted to explore normative or specific (ideographic) prediction models that are available for individuals. Allen et al. [26] developed a short-term prediction model of suicidal thoughts and behaviors, but it was applied to adolescents. Kyron et al. [27] found that short-term fluctuations in self-reported mental health may indicate when an individual is at risk of self-harm. However, the final model in that study only showed acceptable predictive performance when standard logistic regression was performed, with slightly lower sensitivity (71.4%), specificity (77.8%), and positive predictive value (23.9%) statistics. Khazanov et al. [28] conducted a study on the role of distress in predicting treatment outcomes of depression and found that assessing distress before treatment may help determine which patients would benefit most from adding cognitive therapy to antidepressant medications.

These findings supported the generic model and the implication which could be used as a basis to formulate and treat multiple presenting mental problems. Unfortunately, assessing distress and mental risk factors might not have fully captured aspects of one’s mental health [29]. As Fernandez et al. [30] stated, it was possible to develop an algorithm with good discrimination for the onset identifying overall and modifiable risks of common mental disorders among working men, but it was a secondary analysis of the study. Recently, Van Hoffen et al. [31] included distress in a multivariable prediction model for mental long-term sickness absence (LTSA), but the external validation showed that the model may need further improvement due to its not high AUROC value. Compared with these studies, the variables included in this study were new, more comprehensive, and more representative with good prediction ability, and the formula had a good fitting on the medical graduate students.

An example of how to use the formula was given in the following. If a professional-oriented student (2 points) was in the third year (3 points), spent 6 to 10 h (2 points) on scientific research daily with a monthly income of less than 1000 CNY (1 point), and had a mild feelings of time stress (2 points), and his or her scientific learning style was guiding by others (1 point), then the predicted probability of severe mental distress was: P(Y = 1) = e^x^ / (1+ e^x^)= *e*^−1.76^ / (1+ *e*^−1.76^) =10.00%, (x = − 5.06 + 0.20* 3 + 0.44*2 + 0.51*2–0.28* 1 + 0.30*1 + 0.39*2 = −1.76). It meant that there was a 10% probability that the student might suffer from severe mental distress and this student can be regarded as a moderate risk of severe mental distress according to our classifications.

### Limitations and implications

The developed formula had both practical and theoretical implications. On one hand, it enriches and develops the findings of psychological factors linked to stress and severe mental distress in prior studies. On the other hand, it provides a new way for medical graduate students to get a self-report and deal with the possible mental distress in advance.

Several limitations exist even though the current formula may be a promising prediction model of mental distress. The snowball sampling, widely used in cross-sectional studies, is a chain-referral method where initial participants (seeds) recruit others from their social network. But the snowball sampling and the media coverage cannot provide the actual number of the total participants that the survey reached and thus the response rate was not clear to us. Both the Cronbach’s α of the GAD-7 and PHQ-9 revealed the psychological evaluation was reliable in this study, but the reliability of the other parts of the questionnaire still needs to be improved by a deliberate selection of more comprehensive potential predictors of mental distress. As previous researches indicated, mental distress might be affected by gender [32]. Moreover, the influence of personality traits also played an important role in mental health among medical staff [33]. All these variables warranted further consideration and exploration in the future. In addition, the formula still needs external validation while an internal validation was performed in this study. Finally, we surveyed during the Coronavirus disease 2019 (COVID-19) pandemic and the study has shown that the pandemic may also affect individuals’ mental health [34], thus the formula’s applicability in non-pandemic time needs further investigations.

## Conclusions

This study is the first that develops a model to predict mental distress among medical graduate students in China. According to the results, the formula showed relative good discrimination and calibration ability that could identify students with high risks of mental distress. Since timely screening and proper intervention were urgent among Chinese medical graduate students, this formula has the potential to be highly recommended to educational programs, mental health organizations, and especially students with stigma for professional counseling.

## Supplementary Information

**Additional file 1.** Questionnaire on mental distress among medical graduate students of China.

## Data Availability

The datasets generated and/or analyzed during the current study are not publicly available due to limitations of ethical approval involving the participants’ data and anonymity but are available from the corresponding author on reasonable request.

## Abbreviations

WHO: World Health Organization
GAD-7: Generalized Anxiety Disorder Scale-7
PHQ-9: Patient Health Questionnaire-9
DSM-IV: Statistical Manual of Mental Disorders
SCL-90: Symptom check list-90
BDI: Beck Depression Inventory
LASSO: Least Absolute Shrinkage and Selection Operator
AUROC: Area under the receiver operating characteristic curve
LTSA: Long-term sickness absence
CI: Confident interval
CCR: Correct classification rate
POS: Positive
NEG: Negative
CNY: Chinese Yuan
SCI: Science Citation Index
CAS: Chinese Academy of Sciences
CAE: Chinese Academy of Engineering
NSFC: National Natural Science Foundation of China
COVID-19: Coronavirus disease 2019
