# Supplementary material for: A novel model to predict mental distress among medical graduate students in China
Source: BMC Psychiatry. 2021 Nov 15;21:569. doi: 10.1186/s12888-021-03573-9 (PMC8591601; doi:10.1186/s12888-021-03573-9)
Supplement: Supplementary file 1 — Additional file 1. Questionnaire on mental distress among medical graduate students of China. [file 12888_2021_3573_MOESM1_ESM.doc]

**MENTAL DISTRESS AMONG MEDICAL GRADUATE STUDENTS OF CHINA CONSENT FORM**

Dear Participants,

We sincerely invite you to participate in this study entitled ‘*Mental distress among Medical Graduate Students in China*’. This study aimed to investigate the psychological status among medical graduate students in China, to develop a novel model to predict mental distress among those students.

The questionnaire contains about 20 questions and it takes 3 to 5 minutes to complete. This study is conducted online anonymously without obtaining any personally identifiable information. Data from this study will eventually be reported as a whole in academic journals.

You are entirely voluntary to take part in this survey and you have the right to refuse and terminate the investigation at any time for any reason. We would respect your choices if you are reluctant to participate. Please fill out the questionnaire according to your real conditions to ensure that the data are reliable. If you have any questions, please feel free to contact us at [guofeihclgxs163@163.com](mailto:1181130880@qq.com).

Completing this survey indicates that you are 18 years of age or older and indicates your consent to participate in the research.

Thank you for your assistance in this important endeavor.

Sincerely yours,

Fei Guo on behalf of all investigators.

**Subject’s Statement**

🞎  I have read and understood the informed consent and volunteer to participate

in the survey.

🞎  I decline to participate in this study.

**How Are We Doing?**

Please take a few minutes to fill out this survey. Your answers will be kept confidential. Thank you for your participation.

1. You are years old.

**Socio-Demographic Information**

2. Which year are you studying for your degree?

🞎 First year

🞎 Second year 

🞎 Third year

🞎 Fourth year

🞎 In the deferment period

3. What is your major?

🞎Obstetrics and gynecology

🞎Surgery

🞎 Internal Medicine

🞎Basic Medicine

🞎Others

4. Where is your school?

🞎Beijing

🞎Out of Beijing

5. What is your marital status?

🞎Single

🞎In love

🞎Married without childbearing

🞎Married and completed childbearing

6. What is your disposable income monthly (CNY)?

🞎 ＜1000

🞎 1000-3000

🞎 3000-5000

🞎 ＞5000

7. You are a medical student of _____

**Academic performance**

🞎 Master candidate

🞎 Ph.D. candidate

🞎 Postdoctoral candidate

🞎 Seven-year program

🞎 Eight-year program

8. Your school is in the national "Double First-rate" strategic plan

🞎 YES

🞎 NO

9. Your degree is _____.

🞎Research oriented

🞎Professional oriented

10. You are engaged in_____.

🞎 Mainly basic scientific research

🞎 Uncertain

🞎 Mainly clinical research

🞎 Both basic scientific research and clinical research

11. How many hours are you engaged in the above research activities every day?

_____ (ranging from 1 to 24 Hours).

12. How many papers have you published as the first or co-first author (including

English and Chinese papers)?

🞎 1

🞎 2

🞎 3

🞎 4

🞎 ≥5

13. What is the total impact factor are your published papers?

🞎 0

🞎 0-3

🞎 3-6

🞎 6-10

🞎 ≥10

14. How many research projects have you participated in?

🞎 0

🞎 1

🞎 2

🞎 3

🞎 ≥4

15. Who is the main director in your scientific research?

🞎 My tutor

🞎 The associate supervisor

🞎 Senior schoolmates or classmates

🞎 Self-learning

🞎 Others

16. Do you often feel the urgency of time in scientific research?

(1 means none, 2-3 mild, 4-5 moderate, and 6-7 severe)

🞎 1

🞎 2

🞎 3

🞎 4

🞎 5

🞎 6

🞎 7

17. Does your tutor have the following administrative jobs?

**Incumbency of tutor**

🞎 Administrative leader of the hospital

🞎 Administrative leader of the department

🞎 National academician

🞎 Winner of the National Outstanding Youth Science Fund

🞎 The Yangtze River Scholar

🞎 Leader of the Chinese Medical Association /Chinese Medical Doctor

Association

🞎 Others

18. Did your tutor win the National Natural Science Foundation of China in the past five years?

🞎 YES

🞎 NO

**Psychological evaluation**

19. Have you ever diagnosed with anxiety or depression in the hospital?

🞎 YES

🞎 NO

20. Generalized Anxiety Disorder Scale-7 (GAD-7)

| **Over the last 2 weeks, how often have you been bothered by the following problems?** | **Not at all** | **Several days** | **More than half the days** | **Nearly every day** |
| --- | --- | --- | --- | --- |
| **a.** Feeling nervous, anxious, or on edge | 🞎 | 🞎 | 🞎 | 🞎 |
| **b.** Not being able to stop or control worrying | 🞎 | 🞎 | 🞎 | 🞎 |
| **c.** Worrying too much about different things | 🞎 | 🞎 | 🞎 | 🞎 |
| **d.** Trouble relaxing | 🞎 | 🞎 | 🞎 | 🞎 |
| **e.** Being so restless that it is hard to sit still | 🞎 | 🞎 | 🞎 | 🞎 |
| **f.** Becoming easily annoyed or irritable | 🞎 | 🞎 | 🞎 | 🞎 |
| **g.** Feeling afraid as if something awful might happen | 🞎 | 🞎 | 🞎 | 🞎 |

21. Patient Health Questionnaire-9 (PHQ-9)

| **Over the last 2 weeks, how often have you been bothered by any of the following problems?** | **Not at all** | **Several days** | **More than half the days** | **Nearly every day** |
| --- | --- | --- | --- | --- |
| **a.** Little interest or pleasure in doing things | 🞎 | 🞎 | 🞎 | 🞎 |
| **b.** Feeling down, depressed, or hopeless | 🞎 | 🞎 | 🞎 | 🞎 |
| **c.** Trouble falling or staying asleep, or sleeping too much | 🞎 | 🞎 | 🞎 | 🞎 |
| **d.** Feeling tired or having little energy | 🞎 | 🞎 | 🞎 | 🞎 |
| **e.** Poor appetite or overeating | 🞎 | 🞎 | 🞎 | 🞎 |
| **f.** Feeling bad about yourself — or that you are a failure or have let yourself or your family down | 🞎 | 🞎 | 🞎 | 🞎 |
| **g.** Trouble concentrating on things, such as reading the newspaper or watching television | 🞎 | 🞎 | 🞎 | 🞎 |
| **h.** Moving or speaking so slowly that other people could have noticed?  Or the opposite — being so fidgety or restless that you have been moving around a lot more than usual | 🞎 | 🞎 | 🞎 | 🞎 |
| **i.** Thoughts that you would be better off dead or of hurting yourself in some way | 🞎 | 🞎 | 🞎 | 🞎 |
